# An Upstream G-Quadruplex DNA Structure Can Stimulate Gene Transcription

**DOI:** 10.1021/acschembio.3c00775

**Published:** 2024-02-28

**Authors:** Yuqi Chen, Angela Simeone, Larry Melidis, Sergio Martinez Cuesta, David Tannahill, Shankar Balasubramanian

**Affiliations:** † Yusuf Hamied Department of Chemistry, University of Cambridge, Cambridge CB2 1EW, U.K.; ‡ Cancer Research UK Cambridge Institute, University of Cambridge, Cambridge CB2 0RE, U.K.; § School of Clinical Medicine, University of Cambridge, Cambridge CB2 0SP, U.K.

## Abstract

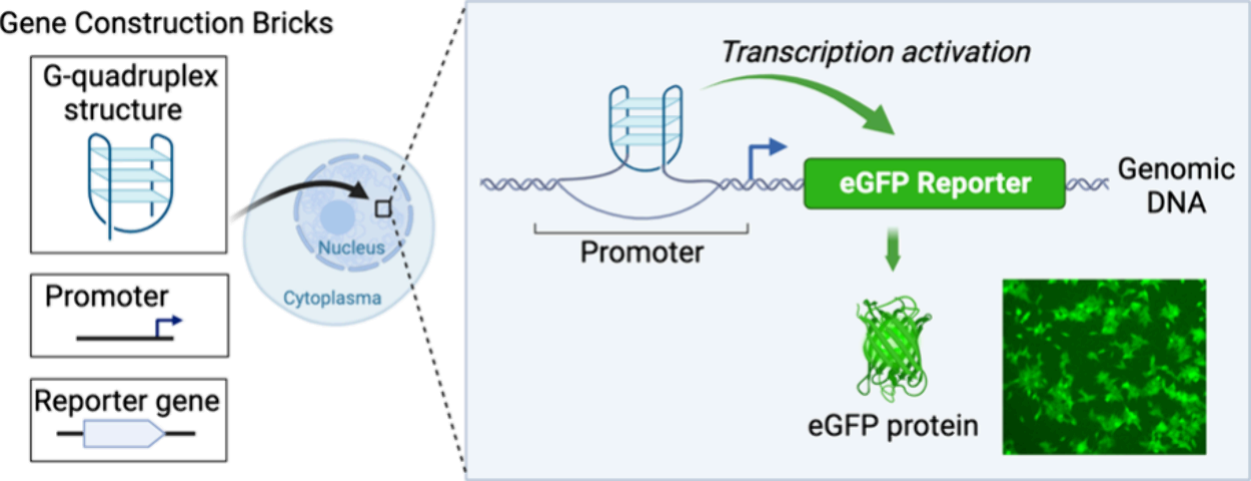

Four-stranded G-quadruplexes (G4s) are DNA secondary structures that can form in the human genome. G4 structures have been detected in gene promoters and are associated with transcriptionally active chromatin and the recruitment of transcription factors and chromatin remodelers. We adopted a controlled, synthetic biology approach to understand how G4s can influence transcription. We stably integrated G4-forming sequences into the promoter of a synthetic reporter gene and inserted these into the genome of human cells. The integrated G4 sequences were shown to fold into a G4 structure within a cellular genomic context. We demonstrate that G4 structure formation within a gene promoter stimulates transcription compared to the corresponding G4-negative control promoter in a way that is not dependent on primary sequence or inherent G-richness. Systematic variation in the stability of folded G4s showed that in this system, transcriptional levels increased with higher stability of the G4 structure. By creating and manipulating a chromosomally integrated synthetic promoter, we have shown that G4 structure formation in a defined gene promoter can cause gene transcription to increase, which aligns with earlier observational correlations reported in the literature linking G4s to active transcription.

## Introduction

**Figure 1:**
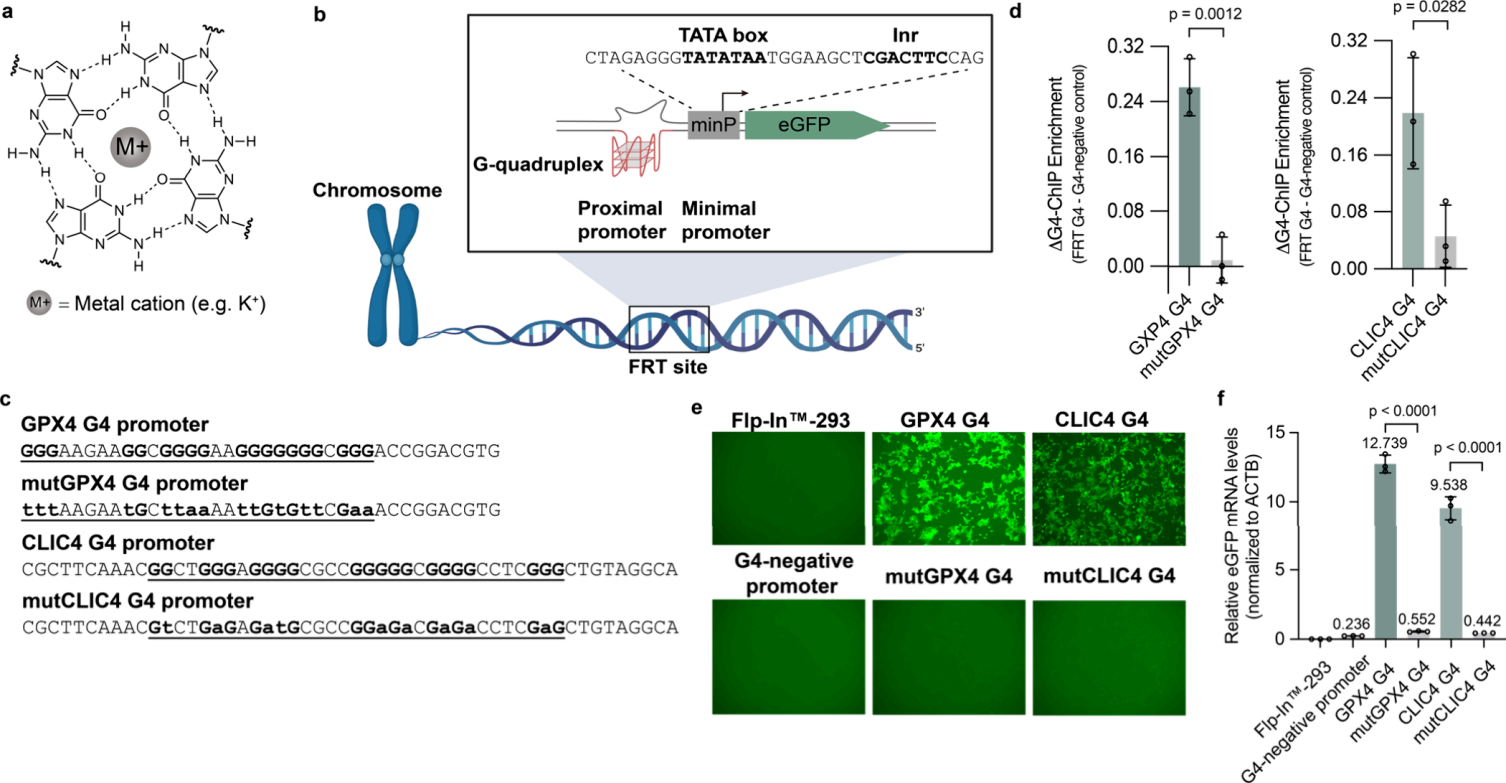
GPX4 and CLIC4 promoter sequences fold into G4 structures in cells and promote eGFP transcription. (a) G-tetrad stabilized by Hoogsteen base pairing and a monovalent cation. (b) Architecture of the G4 promoter eGFP reporter system. A G4 is placed at the proximal promoter region on the template strand. A minP sequence containing a TATA box and Inr acts as a core promoter to mediate transcription initiation and is placed between the G4 and eGFP protein coding sequences. The sequences of TATA box and Inr are in bold. Panel (b) was created with BioRender.com. (c) Sequences of GPX4 G4/mutated GPX4 G4 and CLIC4 G4/mutated CLIC4 G4. Point mutations for mutated G4s are indicated by lowercase letters. (d) G4-ChIP-qPCR analysis of G4 formation at the CLIC4 and GPX4 G4 promoters normalized to a positive control G4 in the host genome (RBBP4) and after background signal subtraction. (e) Representative fluorescence microscopy images of Flp-In expression cell lines showing that promoter GPX4 and CLIC4 G4s drive eGFP protein expression. (f) Quantification of eGFP RNA expression by RT-qPCR. G4-negative promoter: the Flp-In-293 expression cell line in which G4 is absent from the eGFP promoter. Flp-In-293: the parental Flp-In-293 cell line without the integration of the eGFP reporter (mean ± s.d.; two-tailed unpaired *t* test).

Certain guanine-rich DNA sequences can fold into intramolecular fold-back four-stranded secondary structures called G-quadruplexes (G4s).^1^ G4s comprise stacked, Hoogsteen hydrogen-bonding G-tetrads that can be stabilized by a centrally coordinated cation (Figure 1a), with connecting loops of varying lengths and sequences that can be arranged in different orientations. Intramolecular G4s can be thermally stable under conditions of physiological salt, pH, and temperature. Computational prediction^2,3^ and a G4-sensitive DNA sequencing approach^4^ have suggested the potential for folded G4 structures to form at hundreds of thousands of sites in the human genome. Methods developed to detect G4 structures in cellular chromatin have observed only thousands of G4 structures in human cells,^5−9^ suggesting that chromatin context suppresses the folding of most G4 structures. Computational prediction, sequencing experiments, and chromatin mapping approaches all show the enrichment of G4s immediately before the transcription start site (TSS) of many protein coding genes in gene regulatory regions called promoters.^3,10,11^

Multiple lines of evidence highlight promoter G4s as having biological importance for transcription. In vitro, G4s forming at a transcribed gene can stall RNA polymerase transit.^12,13^ Cellular studies using transfected plasmid gene constructs suggest that natural G4 motifs from oncogene promoters, such as MYC, KRAS, and KIT, can modulate transcription of a downstream reporter gene, as compared to mutated G4 motifs.^14−18^ Transcription of VEGF and KRAS oncogenes is also stimulated by promoter G4 folding arising from oxidative damage to DNA bases in promoters.^19,20^ The addition of G4-stabilizing small molecules to cells can alter expression of oncogenes, such as SRC, BCL2, and KIT, and also induce DNA damage to promote cell killing.^21−23^ Detection of folded G4 structures with structure-specific probes shows that folded G4s are consistently detected within accessible chromatin of human cells and their incidence correlates with active gene transcription.^5,24−26^ Furthermore, the differentiation of human stem cells into defined lineages revealed that the dynamic alteration of where folded G4 structures were retained, gained, or lost had a positive correlation with transcriptional activity, active histone marks, and open chromatin.^24^ Proteins involved in the regulation of gene expression, including several transcription factors, can directly bind G4 structures with high affinity.^26−30^ Collectively, such studies lend support for the formation of promoter G4s as positive regulators of transcription.

There are limitations in the existing data that link G4s to transcription. Interpretations rely largely on correlations. Many studies did not include the actual detection of folded G4s and rely on inference from the G4 sequence motifs. Plasmid-based studies do not position the G4 structure within the native locus in a natural chromatin context. Studies from our own laboratory and other laboratories have shown that G4-targeting small molecules can lower transcription at genes with G4 motifs in promoters. A possible interpretation of such studies is that stabilization of G4s inhibits transcription, and therefore, G4s are negative regulators of transcription. There are several reasons to be cautious about interpreting such experiments. Small molecules can bind to thousands of folded G4 targets in the cellular genome with many potential downstream effects. Small molecules can compete off G4-bound proteins (e.g., transcription factors),^28^ and so a small molecule-G4 complex is not just a more stable G4. Last, many small molecules that target G4s, for example, pyridostatin, cause proximal DNA strand cleavage and a downstream cellular DNA damage response,^22^ which confounds a simple interpretation of transcriptional changes in a single gene. Thus, small molecule experiments cause complex effects that can preclude a clear and reliable interpretation. There is a need for studies that can more clearly and directly resolve the relationship between a promoter G4 and transcription.

Herein, we describe the design and assembly of a synthetic promoter-reporter gene regulatory system that was exploited to study the consequential effects of a promoter-G4 on transcription. The observations show that the formation of G4 structures in this context positively regulates gene transcription, with transcription levels being related to the intrinsic thermal stability of the G4 structure.

## Results and Discussion

### Experimental Design

We developed a synthetic gene regulatory construct to directly evaluate the relationship between G4s in the promoter and transcription (Figure 1b,c). Using the Flp-In-293 human cell line, we placed G4 sequences, which are in front of a minimal core promoter (minP) construct that drives the expression of a green fluorescent protein (eGFP) reporter (see Supplementary Methods) into human chromatin. The G4 sequences were always positioned on the template strand. Cell lines were generated by integrating each construct into the same genomic position by site-directed recombination (Supplementary Methods) and confirmed to have a single copy of the correct sequence by Sanger DNA sequencing (Supplementary Methods, Supporting Information).

G4 folding of test sequences was detected and quantified using a folded-G4 structure-specific antibody BG4^25^ to affinity-capture folded G4 structures from isolated chromatin,^5^ followed by quantitative polymerase chain reaction (G4 ChIP-qPCR) using primers adjacent to the G4 site. Fluorescence microscopy or flow cytometry (see Supplementary Methods) of live cells by detecting eGFP fluorescence was used to estimate expression levels. Accurate quantification of transcription was achieved from isolated RNA by quantitative reverse transcription qPCR (RT-qPCR) with gene-specific primers for eGFP using actin expression as a reference.

### Selection of Natural Promoter G4s for Insertion

Experimental mapping of folded G4s in human cell lines^31^ shows that promoter G4s are enriched in front of the transcription start sites of actively transcribing genes (see Supplementary Methods and Figure S1). Glutathione peroxidase 4 (GPX4) and chloride intracellular channel 4 (CLIC4) G4s were selected as representative single G4s present in the proximal promoters of genes undergoing high transcription (see Supplementary Methods and Figure S2). Circular dichroism (CD) spectroscopy^32,33^ confirmed that GPX4 and CLIC4 G4 oligonucleotides fold into structures consistent with parallel (∼265 nm maxima and ∼245 nm minima) or hybrid (∼295 nm, ∼265 nm maxima and ∼245 nm minima) G4 conformations, respectively (Figure S3a,c).^34−36^ Folded G4 formation was disrupted either when tetrad-forming G bases were mutated or when folded in 100 mM LiCl. UV thermal melting spectroscopy^37,38^ indicating that GPX4 and CLIC4 G4s have high thermal stability with melting temperatures (*T*_m_) of 73.5 and 81.0 °C, respectively (Figure S3b,d).

### Promoter G4s Stimulate Transcription

We assessed whether the insertion of GPX4 or CLIC4 G4 sequences, or their mutated variants, causes folded G4 structures in our cell lines as determined by G4 ChIP-qPCR. The RBBP4 G4, situated elsewhere in the genome, was used as a positive reference for G4 folding, and background correction was performed by subtracting the signal detected from cells carrying only the minimal promoter without the G4 insertion. Cells with the GPX4 or CLIC4 G4 sequence exhibited a detectable, folded G4 structure, which was lost upon mutation of critical G bases (Figure 1d). We have therefore created detectable folded G4 structures at specific sites by inserting G4 sequences into the cellular human genome.

We then investigated whether G4 formation was linked to active transcription. For this, we quantified eGFP expression driven from GPX4 or CLIC4 G4-containing promoters compared to mutated promoters unable to form a G4. Fluorescence microscopy of live cells revealed that cells with either a GPX4 or CLIC4 G4 promoter had easily detectable eGFP fluorescence compared to those lacking a G4 or carrying a mutated G4 (Figure 1e). With expression analysis by reverse transcription followed by quantitative polymerase chain reaction of cDNA (RT-qPCR), cells with a GPX4 or CLIC4 G4 showed more than a 40-fold increase in eGFP mRNA compared to cells with only the minimal promoter reporter lacking a G4 (Figure 1f). A similar G4-driven increase (70-fold) in expression was observed when we measured the mean fluorescence intensity (MFI) for eGFP by flow cytometry (Figure S4). Therefore, in our synthetic regulatory system, the addition of a folded G4 structure in the proximal promoter stimulates transcription. When we generated cells carrying a CLIC4 G4 but lacking the minimal core promoter, eGFP reporter expression was ∼90% lower than when both components were present (Figure S5).

The combination of a minimal core promoter and G4 is thus required for maximal transcriptional output.

To rule out whether the activation of gene expression in the G4-positive cell lines is due to intrinsic duplex G-richness, we shuffled the CLIC4 G4 sequence to break up the G4 motif but preserve the overall nucleotide composition. We confirmed that the oligonucleotide for the shuffled G4 did not fold into a G4 structure, as judged by CD spectroscopy (Figure S6a,b). Insertion of the shuffled sequence into the proximal promoter in cells led to a loss of eGFP reporter expression by over 97% to basal levels compared to CLIC4 G4-containing cells (Figure S6c–e). These findings demonstrate that it is the folded G4 structure rather than intrinsic G-richness that stimulates transcription.

### G4 Stimulate Transcription Independent of the SP1 Consensus Motif

**Figure 2:**
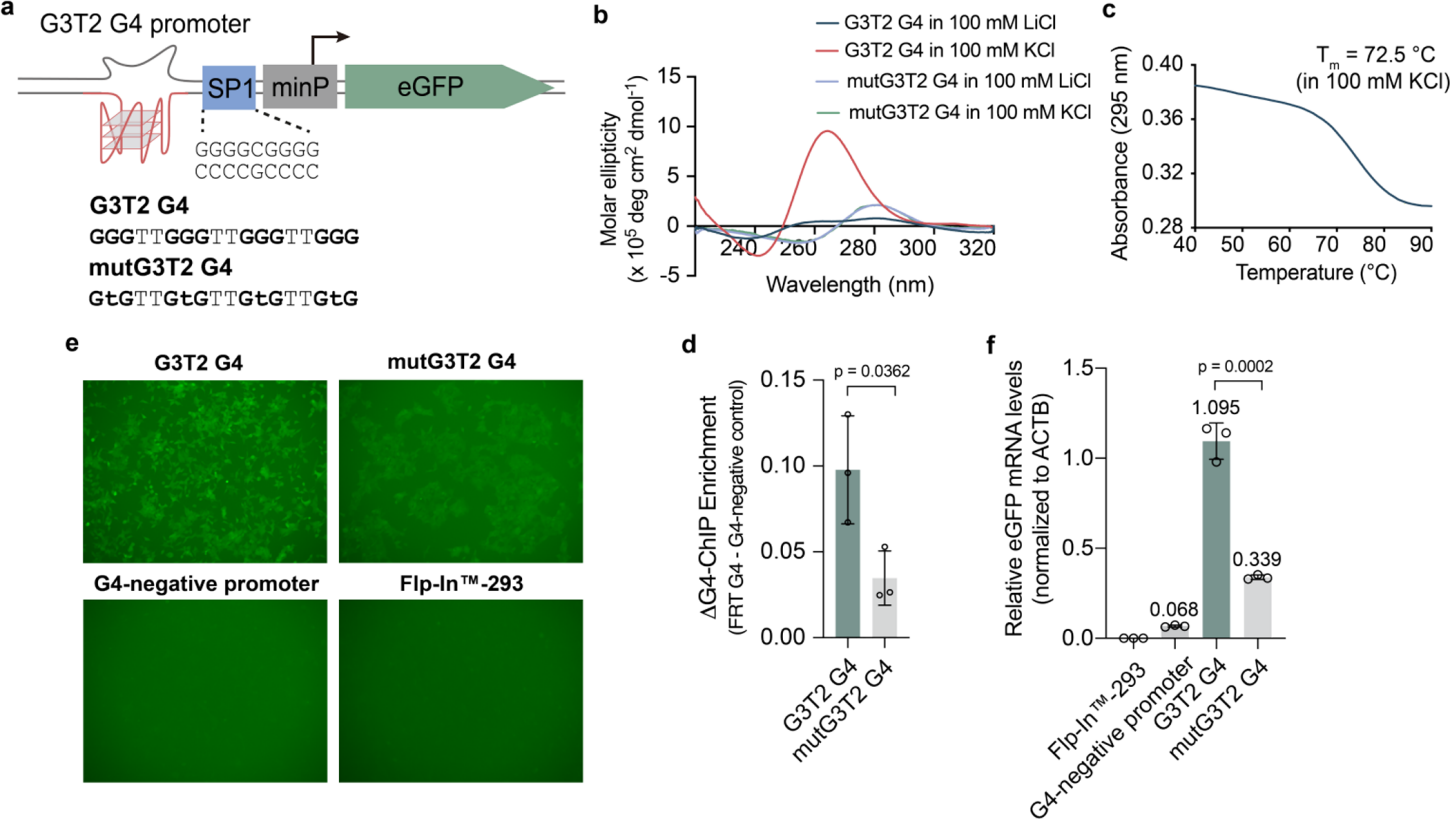
A G4 structure lacking SP1 consensus sequences still promotes enhanced eGFP expression. (a) Architecture of the eGFP reporter system. A synthetic (G3T2)_3_GGG G4 or a mutated version (termed G3T2 or mutG3T2, respectively) is placed in the proximal promoter. A SP1 binding site is also placed between the proximal and core promoters. (b, c) Biophysical characterization of the G3T2 G4 structure. Left, circular dichroism spectroscopy for G3T2 or mutG3T2 G4 oligonucleotides showing spectra characteristic of a G4 structure for G3T2 but not mutG3T2 (buffer conditions: 10 mM Tris–HCl (pH 7.4) with 100 mM KCl or LiCl; oligonucleotide concentration: 10.0 μM). Right, UV-melting curve for the G3T2 G4 oligonucleotide. *T*_m_ is indicated on the graph (buffer conditions: 10 mM Tris–HCl (pH 7.4) with 100 mM KCl; oligonucleotide concentration: 5.0 μM). (d) G4-ChIP-qPCR quantification of G4 formation for G3T2, as described in Figure 1, compared to mutG3T2 cells. (e) Representative fluorescence microscopy images showing that the G3T2 G4 has elevated eGFP protein expression compared to mutG3T2, G4-negative, and host cell line expression. (f) Quantification of the increase in eGFP transcription for G3T2 cells compared to mutG3T2 cells by RT-qPCR as described in Figure 1 (mean ± s.d.; two-tailed unpaired *t* test).

Many G4 sequence motifs overlap with the consensus duplex DNA binding site for the transcription factor SP1, which makes it challenging to distinguish the potential effects of each motif.^39^ We noted that the CLIC4 G4 and GPX4 G4 sequences, described earlier, also contain a SP1 consensus target sequence, which may confound our explanation. We therefore designed a second minimal core promoter system in which a fixed, separate, SP1 consensus sequence was incorporated with an unnatural G4 sequence in front to rule out effects due to simultaneously changing both the G4 and the SP1 binding site (Figure 2a).

We designed a simple G4, (G3T2)_3_GGG (**G3T2**), that excludes motifs for the SP1 canonical duplex binding. Affinity pull-down of the SP1 transcription factor from Flp-In-293 cell lysates using G3T2 G4 and duplex G3T2 (dsG3T2) oligonucleotides and analyzed by western blotting shows that SP1 binds strongly to a folded, but not duplex, G4. As a control, SP1 was shown to bind to G4 structures (G4Myc) and the SP1 duplex consensus (Figure S7). The **G3T2** oligonucleotide folds into a stable G4 structure as assessed by CD spectroscopy and UV thermal melting spectroscopy (*T*_m_ = 72.5 °C) (Figure 2a–c). G4 folding was also abolished in 100 mM LiCl or on mutation of the central tetrad Gs to T ((GTGT2)_3_GTG).

We next measured G4 folding and eGFP expression in cell lines in which either the **G3T2** G4 or the mutated G4 was inserted into the promoter construct (Figure 2a). G4 ChIP-qPCR showed that cells carrying a **G3T2** G4 exhibit a stronger signal for folded G4 compared to cells carrying a mutated G4 motif (Figure 2d). This increase in detectable folded G4 was also accompanied by a ∼3-fold increase in eGFP RNA/protein expression (Figure 2e,f and Figure S4).

These findings confirm that a folded G4 can stimulate transcription independent of the canonical SP1 binding site.

### G4 Stability Can Influence G4 Formation in Cells

**Figure 3:**
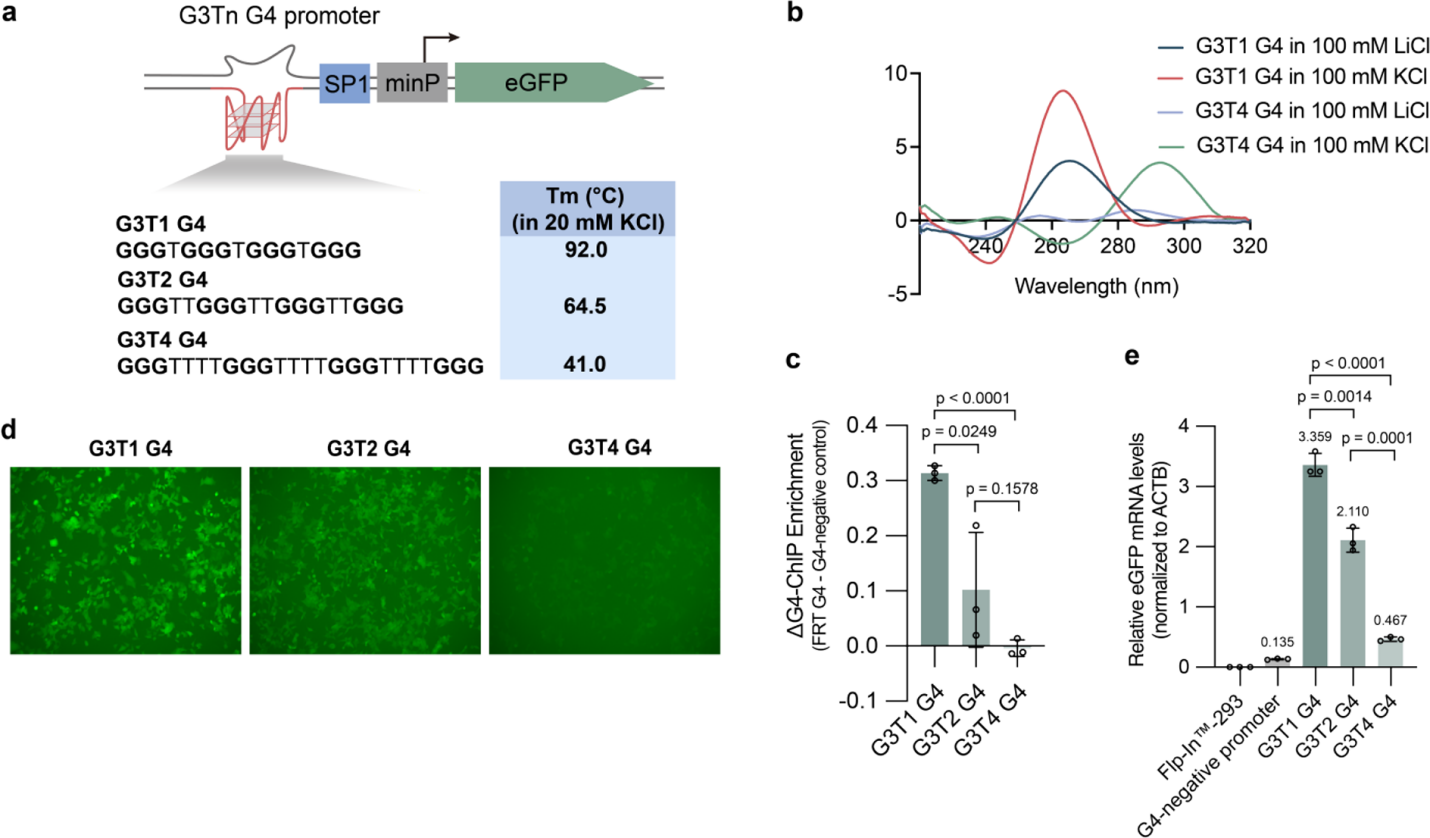
Increased transcriptional activity correlates with increased G4 structural stability in promoters. (a) Sequences of the synthetic G4s G3T1, G3T2, and G3T4 and the corresponding decrease in thermal stability as assessed by oligonucleotide UV melting. (b) Circular dichroism spectra confirming G4 folding for G3T1 and G3T4 in 100 mM KCl or LiCl as per Figure 2 (data for G3T2 is in Figure 2b). (c) Confirmation that increasing G4 stability leads to increased G4 formation in cells, as assessed by G4-ChIP-qPCR. (d) Representative fluorescence microscopy images of the synthetic G4 cell lines showing that eGFP protein expression correlates with G4 stability (data for G3T2 is from Figure 2e). (e) Quantification of the eGFP expression for synthetic G4 cell lines by RT-qPCR showing that expression levels correlate with G4 stability (mean ± s.d.; two-tailed unpaired *t* test.).

We designed a series of unnatural G4s with progressively longer loop sizes of 1, 2, and 4 nucleotides, respectively, (GGGT)_3_GGG (**G3T1**), (GGGTT)_3_GGG (**G3T2**), and (GGGTTTT)_3_GGG (**G3T4**) (Figure 3a), designed to have progressively lower thermal stability based on biophysical studies.^40,41^ G4 folding was verified for each sequence by CD spectroscopy of oligonucleotides^34−36^ (Figures 2b and 3b), and UV thermal melting spectroscopy confirmed that thermal stability decreases with increasing loop length (*T*_m_ = 92.0, 64.5, and 41.0 °C for **G3T1**, **G3T2**, and **G3T4**, respectively (Figure 3a and Figure S8) at 20 mM [K^+^]).

We constructed cell lines with each unnatural G4 sequence inserted in front of the minimal promoter of our assay system with a constant SP1 consensus binding sequence. We then quantified folded G4 folding by G4 ChIP-qPCR. The level of folded G4 folding detected at this site in cells varied systematically with loop length and thermal stability (Figure 3c). The greatest G4 ChIP-qPCR signal was seen in cells with **G3T1**, the signal was reduced in cells with **G3T2**, and no G4 folding was discernible for cells with **G3T4**. In the context of our synthetic cellular system, the extent of folded G4 in cells is therefore directly related to the thermal stability of the G4.

### Increased G4 Stability Leads to Increased Transcription

We then assessed transcriptional output for the **G3T1**, **G3T2**, and **G3T4** cellular insertions by RT-qPCR of eGFP mRNA. The level of eGFP reporter transcription varied systematically with loop length and thermal stability with **G3T1** cells exhibiting the highest transcription, followed by **G3T2** and **G3T4** cells giving ∼1.6- and 9.7-fold lower transcription, respectively (Figure 3d,e). Similar results were obtained using flow cytometry measurement of eGFP fluorescence (Figure S4; ∼1.5- and ∼12.7-fold reduction in MFI for 2- and 4-loop G4s compared to the 1-loop G4). Increased transcription therefore correlates with increased thermal stability of the G4 and the level of folded G4 formation in cells.

## Conclusions

We have presented a systematic study of G4 folding in the context of a synthetic gene promoter that drives a reporter gene in chromatin of human cells. The insertion of natural and unnatural G4 sequence motifs led to a measurable folded G4 structure at the insertion site, with a concomitant increase in transcription of the proximal gene. Systematic variation in the thermal stability of folded G4s, by controlling loop lengths, showed that higher stability gives rise to an increased level of folded G4 signal in cells and higher level of transcription of the reporter gene. The main outcomes support the finding that the presence of folded G4 structures in front of transcribed genes can have a positive effect on transcription, probably through the recruitment of proteins such as transcription factors and chromatin remodelers.

## Supporting Information Available

Detailed description of experimental procedures, materials, and additional figures as mentioned in the text (PDF)
