# Supplementary material for: An Upstream G-Quadruplex DNA Structure Can Stimulate Gene Transcription
Source: ACS Chem Biol. 2024 Feb 28;19(3):736–42. doi: 10.1021/acschembio.3c00775 (PMC10949190; doi:10.1021/acschembio.3c00775)
Supplement: Supplementary file 1 — cb3c00775_si_001.pdf [file cb3c00775_si_001.pdf]

Supporting Information

An upstream G-quadruplex DNA structure can stimulate gene transcription

Yuqi Chen[1], Angela Simeone[1,2], Larry Melidis[1,2], Sergio Martinez Cuesta[1,2], David Tannahill[2], Shankar Balasubramanian[1,2,3*]

[1]Yusuf Hamied Department of Chemistry, University of Cambridge, Cambridge, CB2 1EW, UK

[2]Cancer Research UK Cambridge Institute, University of Cambridge, CB2 0RE, UK

[3]School of Clinical Medicine, University of Cambridge, Cambridge, CB2 0SP, UK

* To whom correspondence should be addressed. Email: SB10031@cam.ac.uk

Containing Supplementary Methods, Materials and Experimental Methods, Bioinformatics data analysis, Scheme S1 and S2, Tables S1 to S6 and Figures S1 to S8.

Supplementary Methods

Validation of single-copy integration of Flp-In expression vectors in the generated expression cell lines

In the Flp-In system, integration of a plasmid into FRT site maintains the FRT site and can result in the integration of additional vector sequences to give unwanted tandem duplications as outlined in Scheme S1.[1] Single vector integration was assessed by qPCR whereby the forward primer was designed at a sequence only present in the FRT locus of the host genome and the reverse primer in the Flp-In expression vector. For multicopy integration testing, primers were designed to the end of the first copy of the integrated vector and at the beginning of the second copy of the vector.

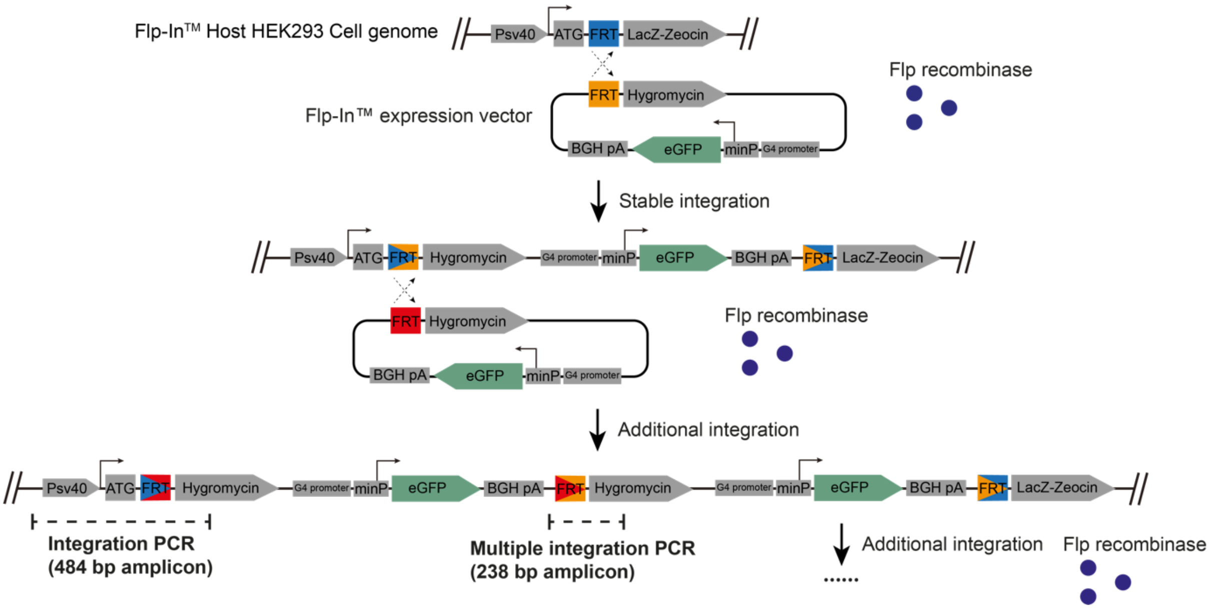

Scheme S1. Illustration of the Flp recombinase-mediated chromosomal integration of genes with multiple copies and the qPCR method to detect integrated copy number. FRT: Flp recombinase recognition target sites. Dashed lines indicate the PCR amplification strategy to discern single and multiple integrations.

Fragments used for the generation of the Flp-In expression vectors

(Template strand sequences are shown for all fragments)

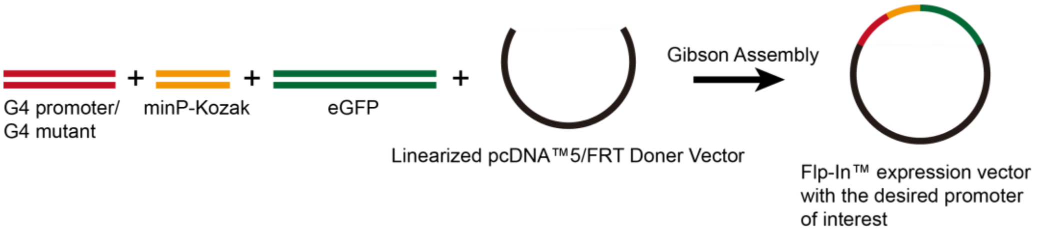

Scheme S2. Diagram of overall structure of G4 promoter Flp-In expression vectors. Promoter-of-interest, minP and eGFP sequences were obtained as described in the Experimental Methods and fused in the intended order into the donor vector to generate the Flp-In expression plasmid. Fragments and their functions are described in more detail below.

G4 promoters and G4 mutants

Bold: G4 motif or G4 mutant

Italic For G3T1, G3T2 and G3T4 G4 promoters, appended SP1 consensus sequence

Bold lowercase: base mutations

Sequences:

CLIC4 G4 promoter sequence:

CCTGCCTACAG**CCCGAGGCCCCGCCCCCGGCGCCCCTCCCAGCC**GTTTGAAGCGGCTCGG

MutCLIC4 G4 promoter sequence:

CCTGCCTACAG**CtCGAGGtCtCGtCtCCGGCGCatCTCtCAGaC**GTTTGAAGCGGCTCGG

GPX4 G4 promoter sequence:

GCACGTCCGGT**CCCGCCCCCCCTTCCCCGCCTTCTTCCC**ACT

mutGPX4 G4 promoter sequence:

GCACGTCCGGT**ttCGaaCaCaaTTttaaGCaTTCTTaaa**ACT

G3T1 G4 promoter sequence:

A**CCCACCCACCCACCC**AGTTTGAA*GGGGCGGGG*

G3T2 G4 promoter sequence:

A**CCCAACCCAACCCAACCC**AGTTTGAA*GGGGCGGGG*

mutG3T2 G4 promoter sequence:

A**CaCAACaCAACaCAACaC**AGTTTGAA*GGGGCGGGG*

G3T4 G4 promoter sequence:

A**CCCAAAACCCAAAACCCAAAACCC**AGTTTGAA*GGGGCGGGG*

minP-Kozak sequence

This contains one TATA box and one initiator element for the initiation of transcription. It also contains one Kozak sequence to initiate protein translation.

Origin: pNL3.2[NlucP/minP] vector (Promega, N1041)

Total: 83 bp

Red: TATA box

Green: Inr

Bold: Kozak sequence (start codon is underlined)

Sequence:

TAGAGGGTATATAATGGAAGCTCGACTTCCAGCTTGGCAATCCGGTACTGTTGGTAAA**GCCACCATGG**

eGFP coding sequence

This codes for the enhanced green fluorescent protein (eGFP).

Origin: pCXLE-EGFP (Addgene, #27082)

Total: 716 bp

Red: stop codon

Sequence:

TGAGCAAGGGCGAGGAGCTGTTCACCGGGGTGGTGCCCATCCTGGTCGAGCTGGACGGCGACGTA

AACGGCCACAAGTTCAGCGTGTCCGGCGAGGGCGAGGGCGATGCCACCTACGGCAAGCTGACCCT

GAAGTTCATCTGCACCACCGGCAAGCTGCCCGTGCCCTGGCCCACCCTCGTGACCACCCTGACCTA

CGGCGTGCAGTGCTTCAGCCGCTACCCCGACCACATGAAGCAGCACGACTTCTTCAAGTCCGCCAT

GCCCGAAGGCTACGTCCAGGAGCGCACCATCTTCTTCAAGGACGACGGCAACTACAAGACCCGCG

CCGAGGTGAAGTTCGAGGGCGACACCCTGGTGAACCGCATCGAGCTGAAGGGCATCGACTTCAAG

GAGGACGGCAACATCCTGGGGCACAAGCTGGAGTACAACTACAACAGCCACAACGTCTATATCAT

GGCCGACAAGCAGAAGAACGGCATCAAGGTGAACTTCAAGATCCGCCACAACATCGAGGACGGC

AGCGTGCAGCTCGCCGACCACTACCAGCAGAACACCCCCATCGGCGACGGCCCCGTGCTGCTGCC

CGACAACCACTACCTGAGCACCCAGTCCGCCCTGAGCAAAGACCCCAACGAGAAGCGCGATCACA

TGGTCCTGCTGGAGTTCGTGACCGCCGCCGGGATCACTCTCGGCATGGACGAGCTGTACAAGTAA

Table S1. DNA oligonucleotides for CD and UV melting assays

Oligomer	Sequence (5' to 3')	Source	Ref
GPX4 G4	GGGAAGAAGGCGGGGAAGGGGGGGCGGG	Sigma-Aldrich	-
mutGPX4 G4	tttAAGAAtGCttaaAAttGtGttCGaa	Sigma-Aldrich	-
CLIC4 G4	GGCTGGGAGGGGCGCCGGGGGCGGGGCCTCGGG	Sigma-Aldrich	-
mutCLIC4 G4	GtCTGaGAGatGCGCCGGaGaCGaGaCCTCGaG	Sigma-Aldrich	-
Shuffled CLIC4 G4	CGGGGGCAGCCCTGGTGTGTCGTCGAGGCGAGCGCATC GGGACGGAGCGGCG	Sigma-Aldrich	-
G3T1 G4	GGGTGGGTGGGTGGG	Integrated DNA Technologies	[2]
G3T2 G4	GGGTTGGGTTGGGTTGGG	Sigma-Aldrich	[3]
G3T4 G4	GGGTTTTGGGTTTTGGGTTTTGGG	Sigma-Aldrich	[3]
mutG3T2 G4	GtGTTGtGTTGtGTTGtG	Sigma-Aldrich	-

Table S2. DNA oligonucleotides for validation qPCRs of single-copy integration

Reaction	Primer	Sequence (5' to 3')	Ref
Integration qPCR	PSV40	AGCTGTGGAATGTGTGTCAGTTAGG	[1]
	Phyr_r	CTTCGCCCTCCGAGAGCTGCATCAG	[1]
Multiple Integration qPCR	PFRT_f	AATCGGGGGCTCCCTTTAGGGTTCC	[1]
	Phyr_r	CTTCGCCCTCCGAGAGCTGCATCAG	[1]

Table S3. DNA oligonucleotides to generate PCR amplicon from the inserted eGFP promoter for Sanger sequencing validation

Primer	Sequence (5' to 3')
YC201-forward	TGCTCTGATGCCGCATAGTT
YC201-reverse	CCAACAGTACCGGATTGCCA

Table S4. DNA oligonucleotides for eGFP-qPCR

Primer	Sequence (5' to 3')	Ref
eGFP-forward	CAGAAGAACGGCATCAAGGTG	-
eGFP-reverse	GGACTGGGTGCTCAGGTAGTG	-
ACTB-forward	AGAAAATCTGGCACCACACC	[4]
ACTB-reverse	TAGCACAGCCTGGATAGCAA	[4]

Table S5. DNA oligonucleotides for G4 ChIP-qPCR

Primer	Sequence (5' to 3')	G4 ChIP	Ref
Flp-In-site-forward	TGGCAATCCGGTACTGTTGG		-
Flp-In-site-reverse	GCTGAACTTGTGGCCGTTTA		-
RBBP4-forward	GAAAGCTACTCCGCGCGTCT	+	[5]
RBBP4-reverse	ACCTTCGCGCCAACATCAG		[5]

Table S6. DNA oligonucleotides for SP1 pull-down and Western Blot analysis

Primer	Sequence (5' to 3')	Ref
3bio_MYC	TGAGGGTGGGTAGGGTGGGTAATTTTT[BtnTg]	[5]
Anti_MYC	TTACCCACCCTACCCACCCTCA	[5]
3bio_G3T2	GGGTTGGGTTGGGTTGGGTTTTTT[BtnTg]	-
Anti_G3T2	CCCAACCCAACCCAACCC	-
SP1-consensus	GATCTTCGAAGTGGGGCGGGGAAGCTTCTAGTTTTTT[BtnTg]	[5]
Anti-SP1-cons	CTAGAAGCTTCCCCGCCCCACTTCGAAGATC	[5]

Materials and Experimental Methods

Oligonucleotides

HPLC purified DNA oligonucleotides were purchased from Integrated DNA Technologies. Oligonucleotides for circular dichroism and UV thermal melting spectroscopy were prepared as previously described.[6] Briefly, oligonucleotides were annealed in 10 mM Tris HCl (pH 7.4) containing 20 or 100 mM KCl, or 100mM LiCl, by heating at 95 °C for 5 min followed by gradually cooling to room temperature. Annealed oligonucleotides were stored at -20 °C. Sequences of oligonucleotides used are given in the supplementary methods.

Circular dichroism spectroscopy

Circular dichroism (CD) spectra were recorded on a Chirascan spectropolarimeter (Applied Photophysics, Leatherhead, U.K.) using a 1 mm path-length quartz cuvette at 25 °C with 10 µM DNA in 200 µL volume. Scans were performed from 320–220 nm, with a 0.3 nm step size, 0.5 s per point and 0.5 nm bandwidth.[6] The recorded spectra were smoothed, subtracted by the blank KCl or LiCl buffer spectra, and zero-corrected at 320 nm.

UV-melting analysis

Temperature-dependent melting curves were measured with a Varian Cary 400 Scan UV-visible spectrophotometer. 600 µL of 5 µM oligonucleotide samples in a stoppered 1 cm path-length quartz cuvette were covered with 600 µL mineral oil and equilibrated at 25 °C for 10 min. Absorbance vs temperature profiles were collected at 295 nm[7] from 25 to 95 °C at a heating rate of 0.5 °C/min. Absorbance values were measured every 0.5 °C. Data underwent non-linear fitting to a Boltzmann equation to calculate the melting temperature (T_m), defined as the temperature at which the folded fraction is 0.5.

Cell culture

Flp-In™ 293 cells (human embryonic kidney cells) (Invitrogen™, R75007) were maintained in high-glucose DMEM (high glucose, with Sodium Pyruvate) (Gibco™, 41966052) supplemented with 10% (v/v) heat-inactivated Fetal Bovine Serum (FBS) (Gibco™, 10082147) and 100 µg mL^{-1} Zeocin™ (Gibco™, R25001) at 37°C. Genetically engineered Flp-In™ 293 cells were grown in high-glucose DMEM supplemented with 10% (v/v) heat-inactivated FBS and 50 µg mL^{-1} Hygromycin B (Thermo Scientific™, 10687-010). Cells were tested periodically for mycoplasma contamination.

Construction of G4 promoter eGFP reporter Flp-In™ expression vectors

Flp-In™ expression vectors were integrated into the Flp-In™ 293 genome at the endogenous FRT site using Flp-mediated recombination. In outline, the expression vectors comprise 4 components: a G4 promoter, a minimal promoter (minP), a green fluorescent protein (eGFP), and the pcDNA5/FRT vector backbone (see Figure 1b, and Supplementary Methods). For the CLIC4 G4 promoter vector, a CLIC4 G4 DNA element was synthesized by Merck & Co., Inc. The minP promoter was PCR amplified from the pNL3.2[NlucP/minP] vector (Promega, N1041). The eGFP reporter was PCR amplified from pCXLE-EGFP (Addgene, #27082). The CLIC4 G4 promoter, minP and eGFP fragments were then cloned into a ApaI linearized pcDNA5/FRT plasmid (Invitrogen™, V601020) by Gibson assembly using the NEBuilder HiFi DNA Assembly Master Mix (NEB, E2621S). The Gibson assembly primers and PCR annealing temperatures were determined using the NEB cloning online assembly tool (NEBuilder® Assembly Tool, accessed at https://nebuilder.neb.com/#!/). The CLIC4 G4 expression vector was then modified to generate other promoter constructs by site-directed mutagenesis to replace or delete promoter elements using the Q5® Site-Directed Mutagenesis Kit (NEB, E0554S). NEBaseChanger® (https://nebasechanger.neb.com/) was used for primer

design and PCR conditions. Mutated G4 promoter vectors were made from the corresponding unmutated vectors. Plasmid vectors were transformed in competent *E.coli* (NEB, C2987H), single clones selected and plasmid DNA prepared by standard methods (NEB, T1010S). Correct vector assembly was confirmed by Sanger sequencing.

Stable Flp-In expression cell line generation

Stable cell lines carrying the desired Flp-In™ expression vectors were generated according to the manufacturer's protocol with minor modifications. In brief, parental Flp-In™ 293 cells were plated into 6-well plates at 0.6 x 10^6 cells per well. The next day, cells were co-transfected with 0.1 mg of the expression vector plasmid DNA and 1.0 mg of the pOG44 Flp-recombinase expression vector plasmid DNA (Invitrogen™, V600520) using 7.5 mL Lipofectamine™ 3000 transfection reagent (Invitrogen™, L3000008). 24 hr after transfection, cells were washed with pre-warmed PBS and add fresh medium to the cells. 48 hr after transfection, cells were plated into 100 mm dishes for selection with 50 µg mL^{-1} Hygromycin B for up to a month. Foci were picked into 96-well plates and 10 to 20 colonies expanded for subsequent validation of the desired recombination event. Validation of correct sequence, integration and copy number was determined by qPCR on isolated genomic DNA from candidate colonies and Sanger sequencing (see Supplementary Methods. Primers are listed in Supplementary Table S2 and S3). Genomic DNA from ~1 x 10^6 cells was isolated using Quick-DNA Microprep Kit (Zymo Research, D3020) as per the manufacturer's instructions. qPCR reactions were performed with 10 µL Fast 2XSYBR Green Master Mix (Thermo Fisher Scientific), 5 µL cDNA diluted 1:50 in nuclease-free water (AM9937, Ambion), and 5 µL 1 µM primer mix, using a C1000 Touch thermal cycler (Bio-Rad, CFX96) with the following program: 95 °C for 20 s, 95 °C for 3 s, 60 °C for 30 s and 70 °C for 1 s, with steps 2–4 repeated for 44 cycles. Melting curves were measured using 0.5 °C step from 65 °C to 95 °C.

eGFP RT-qPCR

Total RNA from cell colonies was isolated by standard TRIzol® protocol (Invitrogen™, 15596026) as per the manufacturer's instructions. 800 ng of total RNA was reverse transcribed using iScript™ cDNA Synthesis Kit (Bio-Rad, 1708890) following the manufacturer's instructions. qPCR reactions were performed as above but with 5.0 µL Fast 2XSYBR Green Master Mix (Thermo Fisher Scientific), 2.5 µL cDNA diluted 1:200 in nuclease-free water (AM9937, Ambion), and 2.5 µL 1 µM primer mix. eGFP expression was normalized to ACTB using the ΔΔCt method. Primer sequences are given in Supplementary Table S4.

G4 ChIP-qPCR/seq

G4-ChIP was used to detect G4 structures in the HEK293 and Flp-In expression cell lines using the structure-specific antibody BG4 as described as previously with the following modifications[8]. Chromatin was isolated from 30 million from each cell line using 250 µL of the Lysis and Hypotonic Buffer for Sonication kit (cat. # 500239, Chromatrap). Lysed nuclei suspensions were sonicated for 30 s on/60 s off (high setting) using a water-cooled bath sonicator (Bioruptor Plus, Diagenode) until genomic DNA was sheared to an average size of 100–500 bp. For each ChIP and input, 3.0 µL of 250 ng μL^{-1} chromatin were incubated in 90.0 µL blocking buffer (25 mM HEPES pH 7.5, 10.5 mM NaCl, 110 m KCl, 1 mM $MgCl_2$ and 1% (w/v) bovine serum albumin; cat. #A7030, Merck) supplemented with 1.0 µL 1 mg mL^{-1} RNaseA (cat. # AM2271, ThermoFisher) for 60 min at 1400 rpm on a ThermoMixer (Eppendorf) at 37 °C in a 1.5 mL DNA LoBind tube. 65 µL anti-flag M2 magnetic beads (cat. # M8823, Merck) were washed 3 times with 650 µL blocking buffer and stored in 1.30 mL blocking buffer at 16 °C on the ThermoMixer (1400 rpm) for at least 1 h until required. Recombinant BG4 antibody was expressed via autoinduction as previously described[9]. For

each ChIP, 1.5 μL of 6.6 μM BG4 stock was added and incubated for 1 h at 16 °C with mixing at 1400 rpm. 2 input samples were processed in parallel with omission of the BG4 antibody immunoprecipitation step. Next, 50 μL of prepared bead solution was added and incubated for 1 h at 16 °C with mixing at 1400 rpm. Beads were magnetically captured, supernatant discarded and washed eight times with 250 μL pre-chilled wash buffer (10 mM Tris pH 7.4, 100 mM KCl, 0.1% (v/v) Tween 20; cat. # 11332465001, Merck) in the cold room. Beads were then resuspended in 250 μL wash buffer and incubated at 37 °C in a ThermoMixer at 1400 rpm for 10 min. After a second warm wash, beads were resuspended in 75 μL of elution buffer (1 x TE buffer, 1 μL 20 mg mL^{-1} proteinase K; cat. # AM2546, ThermoFisher) and incubated at 65 °C with 1400 rpm shaking for 16 h. DNA was purified using MinElute Reaction Cleanup Kit according to the manufacturer's instructions (cat. # 28206, Qiagen) and eluted in 20 μL EB buffer.

For library preparation, samples were quantified by Qubit dsDNA HS assay (ThermoFisher, cat. # Q33230) and 10ng of purified ChIP or input DNA tagmentated (2.5 μL Tn5 (Illumina) in tagmentation buffer (Illumina)) in total reaction volume of 40 μL at 800 rpm shaking for 20 min at 37 °C. After purification by MinElute Reaction Clean up Kit, libraries were generated by mixing 20 μL tagmentated DNA, 2.5 μL each of Nextera Index i7 and Nextera Index i5 (Nextera index primer kit, Illumina) with 25 μL NEB Next High Fidelity 2 x PCR master mix (NEB) in a PCR reaction using the following conditions: 72 °C for 5 min, 98 °C for 30 sec followed by six cycles of 98 °C for 10 sec, 63 °C for 30 sec and 72 °C for 1 min. Libraries were purified using MinElute reaction clean-up kits and library quality/quantity assessed using an Agilent Bioanalyser and Qubit, respectively. Samples were sequences on an Illumina NextSeq platform (single-end, 75 bp reads).

For qPCR, Purified ChIP DNA was diluted 1:6 and analyzed by qPCR with locus specific primers (Supplementary Table S5) as described above. Signal at the locus of interest was normalized to signal for the well characterized RBBP4 positive G4 site[8] in the host cells using the ΔΔCt method. Signal from G4 motif-deleted Flp-In engineered cells was used to calibrate ChIP signal against background. 4 ChIP replicates were performed for each cell sample and the Cq value differing most was discarded.

G4 affinity enrichment and western blotting analysis

Exponentially growing Flp-In^{TM}-293 cells were harvested and lysed at a density of 10 million cells per 300 µL in low salt buffer (20 mM HEPES, pH 7.4, 10 mM NaCl, 3 mM $MgCl_2$, 0.2 mM EDTA, 1 mM Dithiothreitol (DTT) and 1xPIC (complete protease inhibitor cocktail, ThermoFisher Scientific, cat. no. 78429)) on ice for 15 min. Then, 15 µL of 10% w/v NP-40 (ThermoFisher Scientific, cat. no. 85124) were added and pellets vortexed for 1 min followed by centrifugation (900 g, 10 min, 4 °C) to pellet nuclei and washing with low salt buffer. Nuclei were then lysed at a density of 30 million cells per 250 µL in high salt buffer (20 mM HEPES, pH 7.4, 500 mM NaCl, 3 mM $MgCl_2$, 0.2 mM EDTA, 0.5% w/v NP-40, 1 mM DTT and 1xPIC), and sonicated in a Diagenode Bioruptor Plus (ten cycles at 4 °C, each cycle 30 s on and 30 s off at high setting). Lysates were cleared by centrifugation (16,000*g*, 10 min, 4 °C) and supernatant collected. Protein concentration was assessed using BCA protein assay.

A slurry (50 µL) of Streptavidin MagneSphere paramagnetic beads (Promega, cat. no. Z5481) was prewashed with pull-down buffer (25 mM HEPES, 10.5 mM NaCl, 110 mM KCl, 1 mM $MgCl_2$, 0.01 mM $ZnCl_2$, 20% glycerol (v/v), 0.1% Igepal C-630 (v/v), 1 mM DTT and PIC) containing 3% BSA (w/v) and 0.2 g L^{-1} salmon sperm DNA (Invitrogen, cat. no. 15632011) three times (2 ml). 75 µg of nuclear proteins were added to 500 µL pull-down buffer containing 3% w/v BSA (Merck, cat. no. A7030) and 0.2 g L^{-1} salmon sperm DNA, and then precleared

by incubating with the prewashed beads at 4 °C for 2 h. Meanwhile, another 50 µL of beads were washed in the same manner as above. Then, 50 µL of 10 µM annealed biotinylated oligonucleotides (Sigma-Aldrich) were added into 500 µL of pull-down buffer and incubated with the prewashed beads by rotation at room temperature for 30 min. Beads were then washed with pull-down buffer (2 m 3×) and incubated with the precleared lysates (500 µL) by rotation at 4 °C overnight. Beads were next washed with cold pull-down buffer (500 µL 5×) and the bound proteins eluted in 25 µL of LDS sample buffer containing freshly prepared 50 mM DTT by heating at 70 °C for 10 min. Next, 3 µL of the LDS sample buffer were analyzed with capillary electrophoresis in a WES Protein Simple western system with SP1 primary antibody (Proteintech, cat. no. 21962-1-AP) and corresponding secondary antibodies (anti-rabbit). Bands were analyzed using Compass software (ProteinSimple).

Bioinformatics data analysis

G4 ChIP-seq processing and identification of DNA G4 regions

Fastq reads were trimmed from Illumina adapters using cutadapt[10] (-m 10 -q 20 -O 3 -a CTGTCTCTTATACACATCT). Reads aligned to the human genome hg38[11]with bwa mem[12] and alignments were saved into bam files. Picard MarkDuplicates[13] was used to identify and remove duplicated reads. The total number of unique reads was quantified for each library. Regions with local enrichments were obtained by calling peaks with macs2[14] for each individual pull-down library paired to the corresponding input control. Consensus regions of each biological replicate were defined as the regions observed in 2 out of the 3 technical replicates (bedtools, multiIntersectBed[15]).

External public G4 ChIP-seq processing

G4 ChIP-seq data relative to the K562 cell line were downloaded (https://www.ncbi.nlm.nih.gov/geo/query/acc.cgi?acc=GSE107690) and processed similarly to the ones profiling HEK293.

CAGE data - cluster extraction (promoters)

The R package CAGEr (https://bioconductor.org/packages/release/bioc/html/CAGEr.html) was used to download the intended CAGE data. Specifically, the dataset FANTOM5humanSamples was loaded and the recorded IDs relative to K562 and HEK293 fetched for importing the CAGE data. The cumulative distribution of CAGE tags was calculated using the function cumulativeCTSSdistribution (options: returnInterquantileWidth = TRUE, qLow = 0.1, qUp = 0.9). CAGE clusters were identified by using tagClustersGR using all available replicates for each cell line respectively, as well clusters observed across the entire database (all promoters). The clusters with TPM>1 were used for all the subsequent analysis.

Distance analysis using G4 ChIP data and CAGE clusters (promoters)

Distance analysis was conducted as it follows: 1) Selection of the G4 peaks within 5kb to any CAGE promoters (to exclude all G4 peaks not in proximal promoters); 2) For the selected G4 regions, the center of the regions was extracted; 3) The relative distance between G4 peak center and the CAGE promoter was calculated using cell-specific data (G4 HEK293 with CAGE promoter HEK293 and G4 K562 with CAGE promoter K562); 4) For each cell type, the frequency of all distances obtained and used to produce a histogram of distances; the underlying density curve, relative to the obtained histogram, was fitted; 5) The maximum density (y-value) was identified, and the corresponding distance (x-value) extracted and visualized with a vertical red line on the histogram plot. The distance corresponding to the

maximum density value was classed as the most frequently observed distance between the G4 regions and the TSS (CAGE-defined). The distance analysis was conducted using all promoters (all clusters) as well as stratifying clusters, narrow (IQR <= 4bp) or broad (IQR > 4bp). The TSS expression levels for all promoters were obtained, and the proportion of the promoters with or without G4s with expression levels over TPM >= 0.1 was calculated.

Promoter G4 motif selection in human chromatin

The selection of candidate G4s was data-led and made on the basis of publicly available datasets on G4 folding potential and gene transcription. The following HEK293 datasets were integrated using the human genome version hg38 and its associated gene annotations as reference[11]: 1) G4 ChIP-seq signal obtained from our generated G4 ChIP-seq; 2) putative G-quadruplex forming sequences obtained using regular expression matching; 3) observed G-quadruplex sequences obtained from G4-seq experiments as available in GSE110582 (https://www.ncbi.nlm.nih.gov/geo/query/acc.cgi?acc=GSE110582); 4) open chromatin regions extracted from a DNase-seq dataset available in ENCODE's ENCSR000EJR (https://www.encodeproject.org/experiments/ENCSR000EJR/); 5) RNA-seq gene expression data in GSE106476 (https://www.ncbi.nlm.nih.gov/geo/query/acc.cgi?acc=GSE106476).

Most datasets were ready to use upon direct download after minor transformations performed using in-house scripts in the Bash, R and Python programming languages, the genome reference conversion tool LiftOver (https://genome.ucsc.edu/cgi-bin/hgLiftOver) and processing of peak files using bedtools (v2.26.0)[15]. Raw files that required end-to-end processing were RNA-seq datasets. Fastq files were downloaded using the SRA-Toolkit (https://hpc.nih.gov/apps/sratoolkit.html) and sequencing quality checked using FastqQC (https://www.bioinformatics.babraham.ac.uk/projects/fastqc/). Removal of Illumina adapters

and quality trimming was performed using cutadapt[10], reads were aligned to the reference genome using bowtie2[16] and gene expression quantified using RSEM[17]. Finally, gene promoters were extracted from genome reference annotations using the Bioconductor library GenomicFeatures[18].

Integration of the multiple data sources was undertaken with the R library data.table (https://cran.r-project.org/web/packages/data.table/index.html) and bedtools to intersect and merge genomic regions. Data visualizations were performed using the R libraries ggplot2 (https://cran.r-project.org/web/packages/ggplot2/index.html), ggpubr (https://cran.r-project.org/web/packages/ggpubr/index.html) and genome views generated using the Integrative Genomics Viewer IGV[19].

Promoter G4 selection was as follow: 1) selection of G4 ChIP peaks at active promoters (within -1kb upstream of TSSs) associated with elevated transcription. 2) observed G-quadruplexes (OQs) identified by G4-seq[20] as this validates capacity for G4 formation in vitro. G4 peaks overlapping with OQs was chosen. 3) Potential quadruplex sequence motifs (PQSs)[21] were searched within G4 ChIP peaks, and peaks overlapping with PQSs selected. To simplify the selection, G4 ChIP-seq peaks containing only one PQS were selected and a short stretch of DNA bearing this PQS used to construct G4 promoter eGFP reporters. With this approach, the GPX4 and CLIC4 promoter G4s stood out as two top hits and were selected for further experimental validation.

Data availability

The G4 ChIP-seq data of HEK293 cells generated in this study have been deposited to the NCBI Gene Expression Omnibus repository (GEO; https://www.ncbi.nlm.nih.gov/geo/) under the accession code GSE241008.

Code availability

Customized code for G4 ChIP-seq and CAGE is deposited in https://github.com/angsim/G4_stimulate_gene_transcription/tree/main and for the Promoter G4 motif selection is available at https://github.com/semacu/dna-g4-transcription.

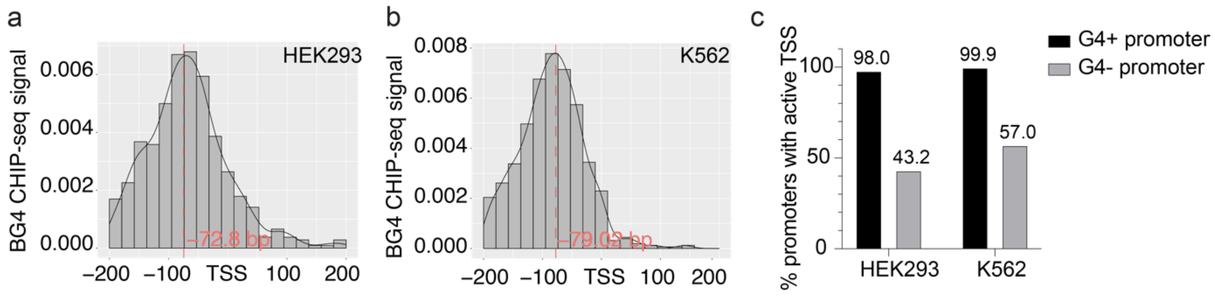

Figure S1. G4s enrichment in the proximal promoter of active genes

(a,b) Genome-averaged density profile of G4 ChIP-seq signal at CAGE-defined promoters (TSS ± 200 bp) in HEK293 cells (**a**) and K562 cells (**b**). The dashed vertical line represents the peak summit, the relative position of G4s (centre of consensus G4 regions, consensus based on 2 out of 3 biological replicates) with respect to the TSS are reported. TSS regions are those based on CAGE signals. Bin size: 20 bp. (**c**) Barplot illustrating the fraction of active promoters belonging to the G4+ and G4-promoter groups. Active promoter is defined as CPM (CAGE-seq) > 0.1 within +/-200 bp from TSS.

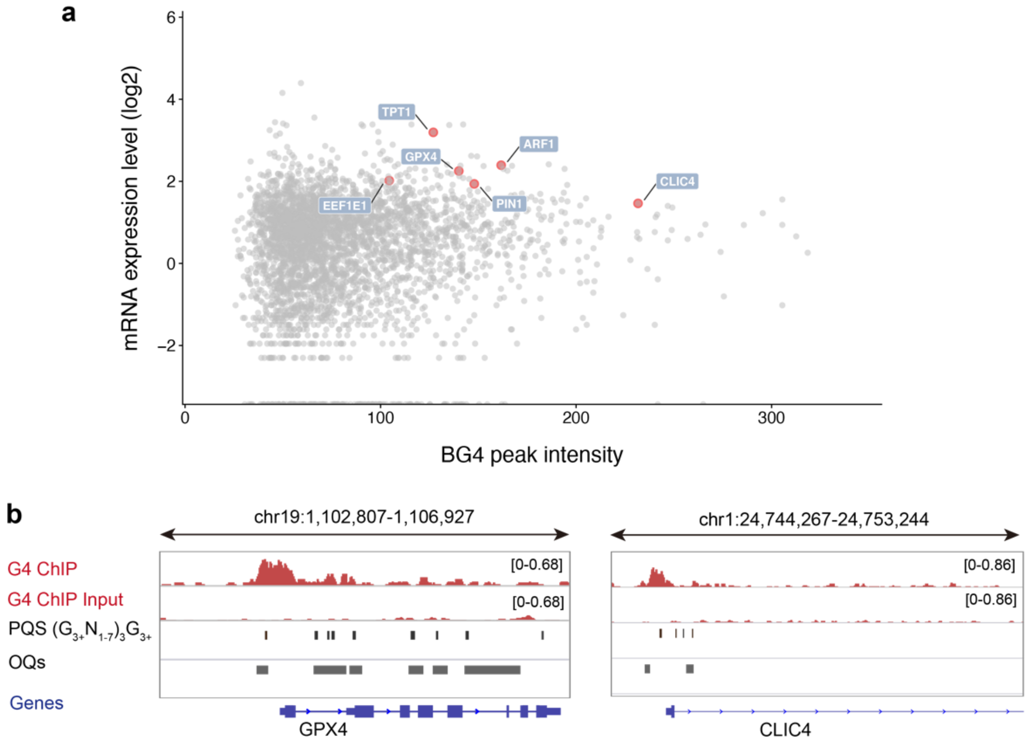

Figure S2. G4 formation relative to RNA expression in HEK293 cells.

(a) Scatter plot of promoter G4 peak intensity versus RNA expression levels. **(b)** Genomic browser view of GPX4 and CLIC4 promoter G4s in HEK293. Top to bottom tracks show G4-ChIP-seq signal, G4-ChIP input control, putative G-quadruplex forming sequences, sequence motifs that can fold into G4 structures *in vitro* (OQs), and gene coding structure.

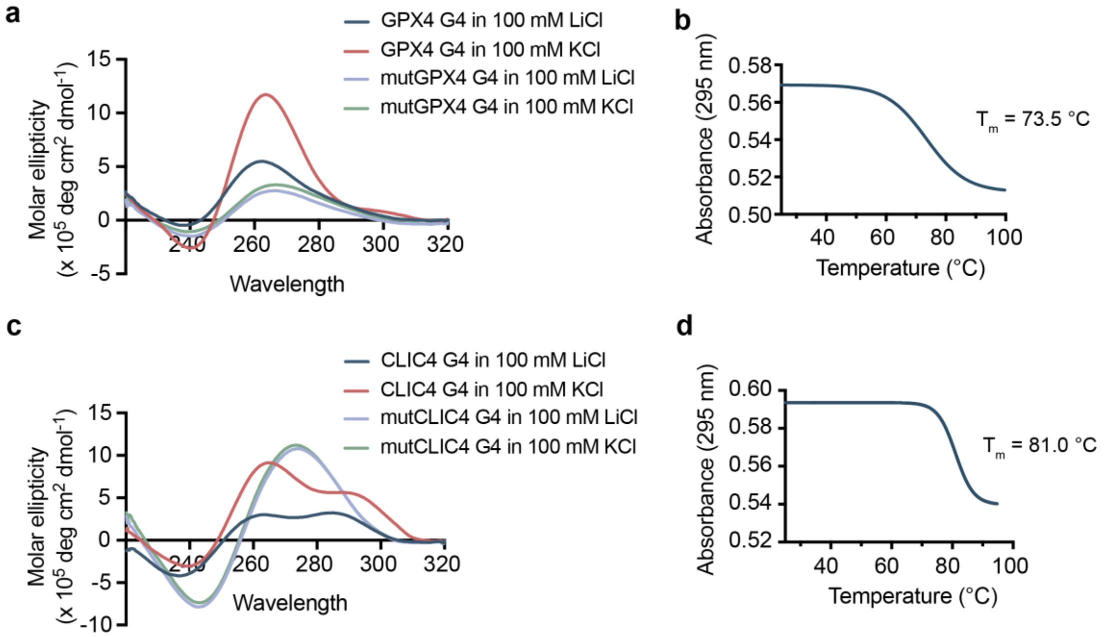

Figure S3. Biophysical characterization of the GPX4 and CLIC4 G4 structures.

(a,c) Circular dichroism spectra of oligonucleotides for GPX4 G4 (**a**) and CLIC4 G4 (**c**) show that both G4s form G4 structures (Buffer conditions: 10 mM Tris-HCl (pH 7.4) with 100 mM KCl or LiCl; oligonucleotide concentration: 10.0 μM). **(b,d)** UV-melting curves showing the high thermal stability of GPX4 G4 (**b**) and CLIC4 G4 structures (**d**). The T_m for each G4 is indicated of the graph. (Buffer conditions: 10 mM Tris-HCl (pH 7.4) with 100 mM KCl; oligonucleotide concentration: 5.0 μM).

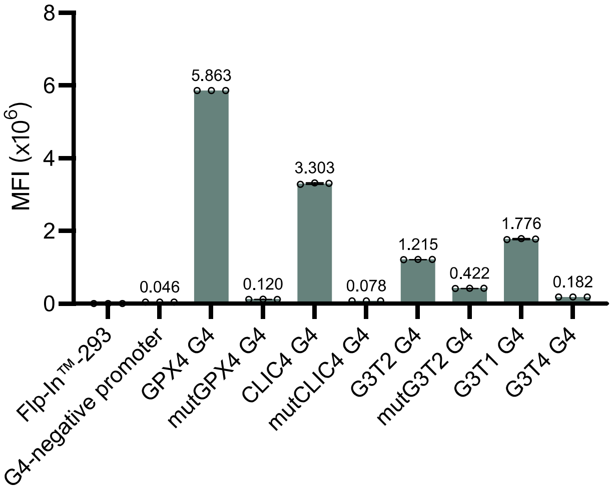

Figure S4. Quantification of eGFP protein expression by flow cytometry for engineered cell lines.

eGFP protein expression in the engineered cell lines described in Figures 1–3 was determined by flow cytometry (see Experimental Methods). Y-axis shows mean fluorescence intensity, values are the mean ± s.d.; two-tailed unpaired *t*-test.

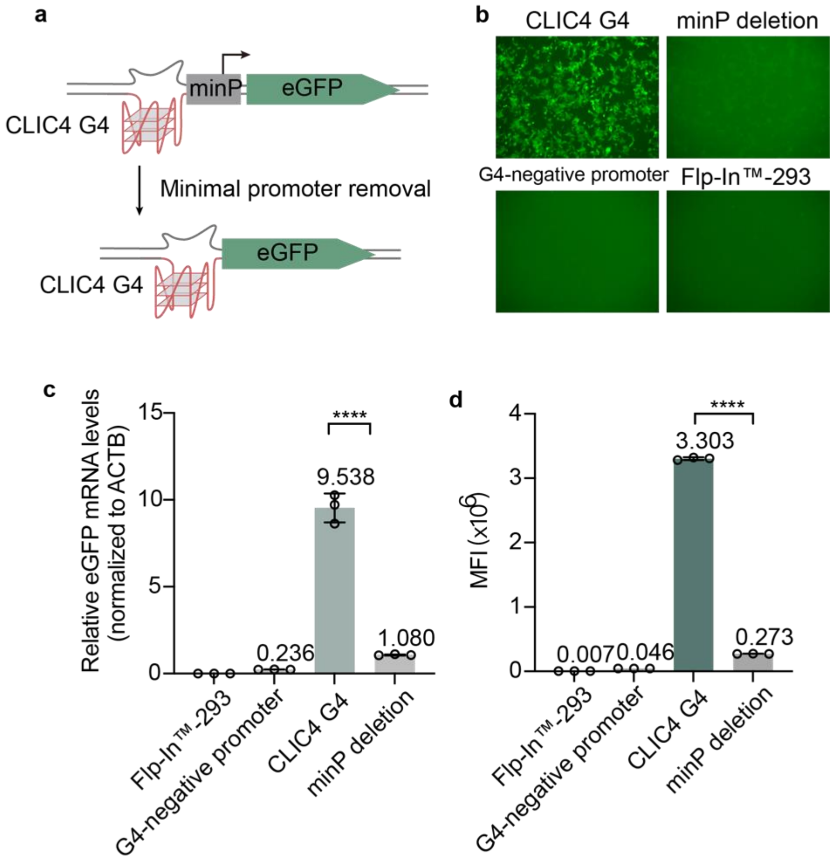

Figure S5. Core promoter deletion abrogates G4 mediated transcription.

(a) Scheme illustrating the core promoter deletion from the CLIC4 G4 promoter eGFP reporter. **(b)** Representative fluorescence microscopy image of the Flp-In^{TM} expression cell lines with CLIC4 G4 promoter and a minP-deleted CLIC4 G4 promoter. eGFP transcription was significant diminished after the removal of minP. **(c,d)** Quantification of the eGFP expression by RT-qPCR (**c**) and flow cytometry (**d**) for constructs lacking minP. As a reference, the data shown for other constructs comes from Figs 1–3 and Figure S4 of this manuscript. (Mean ± s.d.; two-tailed unpaired *t*-test.)

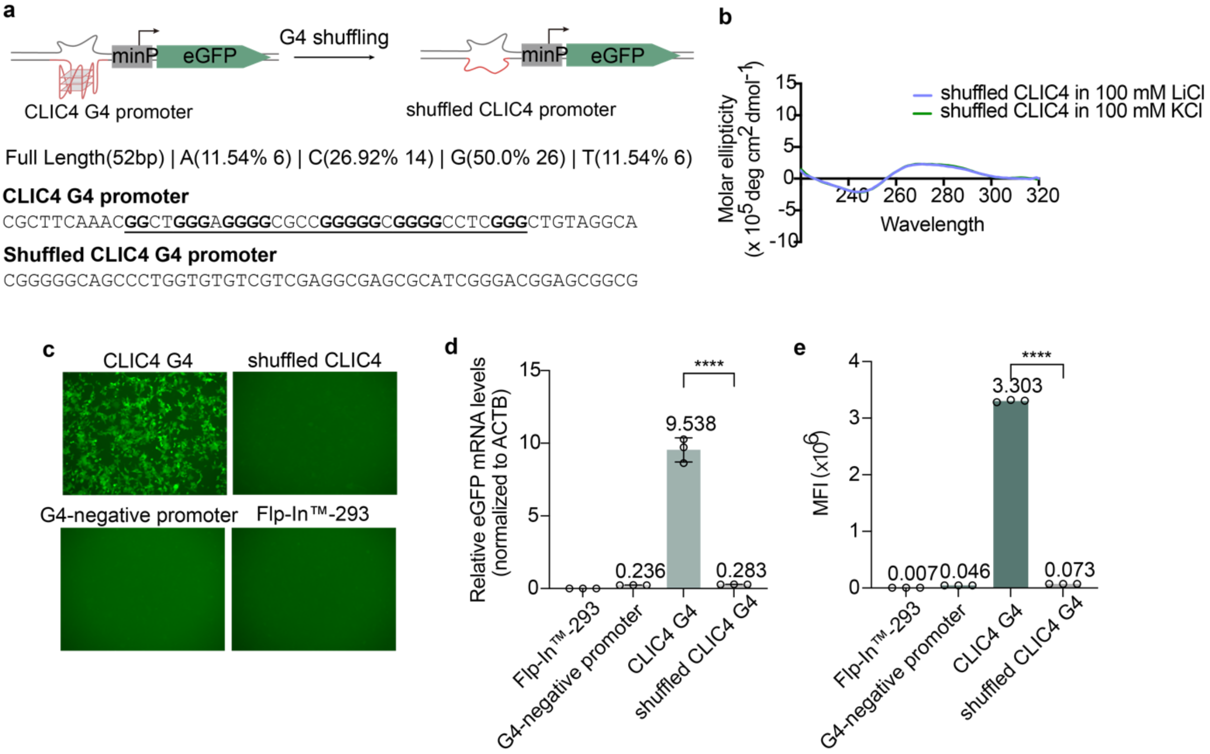

Figure S6. G-richness in the proximal promoter alone does not promote transcription

(**a**) The CLIC4 G4 promoter was shuffled to maintain G-richness and base composition while eliminating motifs that could fold into a G4 structure; (**b**) Circular dichroism spectra of an oligonucleotide of the shuffled CLIC4 G4 shows loss of G4 formation. (**c**) Representative fluorescence microscopy images of the Flp-In^{TM} expression cell lines with shuffled CLIC4 G4 promoter in comparison to that of the CLIC4 G4 promoter and G4-deleted promoter. eGFP transcription was significantly reduced after shuffling the G4. (**d,e)** Quantification of the eGFP expression by RT-qPCR (**d**) and flow cytometry (**e**) for the G4 shuffled construct. As a reference, the data shown for other constructs comes from Figure 1–3 and Figure S4 of this manuscript (Mean ± s.d.; two-tailed unpaired *t*-test.)

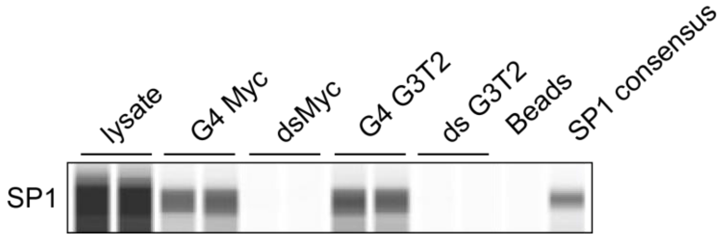

Figure S7. Affinity pull-down of SP1 protein from Flp-In^{TM}-293 cell lysate with G3T2 G4 or G3T2 duplex oligonucleotides. The G3T2 duplex, in contrast to folded G3T2 G4 oligonucleotides, do not enrich SP1 for nuclear lysates. Known G4 Myc and SP1 duplex consensus oligonucleotides were used as positive controls to validate SP1 binding. Lysate: nuclear lysate from Flp-In^{TM}-293 cells, G4 Myc/G4 G3T2: folded Myc/G3T2 G4 structures, dsMyc/dsG3T2: duplex structures formed by Myc/G3T2 oligonucleotides and complementary sequences, beads: bead only control with no immobilized oligonucleotides. SP1 consensus: 5'-GGGGCGGGG-3'/3'-CCCCGCCCC-5'.

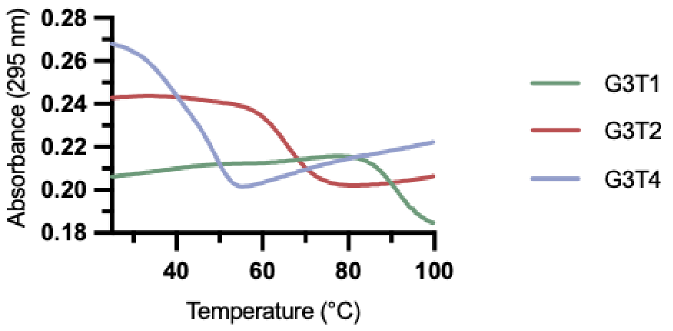

Figure S8. UV melting curves of G3Tn G4s. UV-melting curves showing the thermal stability decreasing with shorter loop of G3Tn G4. The T_m for each G4 is shown in Figure 3a. (Buffer conditions: 10 mM Tris-HCl (pH 7.4) with 20 mM KCl; oligonucleotide concentration: 5.0 μM).

References

(1) Jensen, O., Ansari, S., Gebauer, L., Müller, S. F., Lowjaga, K. A. A. T., Geyer, J., Tzvetkov, M. V., and Brockmöller, J. (2020) A double-Flp-in method for stable overexpression of two genes. *Sci. Rep. 10 (1)*, 1–14.

(2) Bugaut, A., and Balasubramanian, S. (2008) A sequence-independent study of the influence of short loop lengths on the stability and topology of intramolecular DNA G-quadraplexes. *Biochemistry 47*, 689–697.

(3) Guédin, A., Gros, J., Alberti, P., and Mergny, J. L. (2010) How long is too long? Effects of loop size on G-quadruplex stability. *Nucleic Acids Res. 38*, 7858–7868.

(4) Silva Lagos, L., Luu, T. V., De Haan, B., Faas, M., and De Vos, P. (2022) TLR2 and TLR4 activity in monocytes and macrophages after exposure to amoxicillin, ciprofloxacin, doxycycline and erythromycin. *J. Antimicrob. Chemother. 77*, 2972–2983.

(5) Spiegel, J., Cuesta, S. M., Adhikari, S., Hänsel-Hertsch, R., Tannahill, D., and Balasubramanian, S. (2021) G-quadruplexes are transcription factor binding hubs in human chromatin. *Genome Biol. 22*, 1–15.

(6) Zhang, X., Spiegel, J., Martínez Cuesta, S., Adhikari, S., and Balasubramanian, S. (2021) Chemical profiling of DNA G-quadruplex-interacting proteins in live cells. *Nat. Chem. 2021 13:7 13*, 626–633.

(7) Mergny, J. L., and Lacroix, L. (2009) UV Melting of G-Quadruplexes. *Curr. Protoc. Nucleic Acid Chem. 37*, 17.1.1-17.1.15.

(8) Hänsel-Hertsch, R., Beraldi, D., Lensing, S. V., Marsico, G., Zyner, K., Parry, A., Di Antonio, M., Pike, J., Kimura, H., Narita, M. et al. (2016) G-quadruplex structures mark human regulatory chromatin. *Nat. Genet. 48(10)*, 1267–1272.

(9) Biffi, G., Tannahill, D., McCafferty, J., and Balasubramanian, S. (2013) Quantitative visualization of DNA G-quadruplex structures in human cells. *Nat. Chem. 5(3)*, 182–186.

(10) Martin, M. (2011) Cutadapt removes adapter sequences from high-throughput sequencing reads. *EMBnet. J. 17*, 10–12.

(11) Frankish, A., Diekhans, M., Jungreis, I., Lagarde, J., Loveland, J. E., Mudge, J. M., Sisu, C., Wright, J. C., Armstrong, J., Barnes, I. et al. (2021) GENCODE 2021. *Nucleic Acids Res. 49*, D916–D923.

(12) Li, H., and Durbin, R. (2009) Fast and accurate short read alignment with Burrows–Wheeler transform. *Bioinformatics 25*, 1754–1760.

(13) Picard Tools - By Broad Institute.

(14) Zhang, Y., Liu, T., Meyer, C. A., Eeckhoute, J., Johnson, D. S., Bernstein, B. E., Nussbaum, C., Myers, R. M., Brown, M., Li, W., and Shirley, X. S. (2008) Model-based analysis of ChIP-Seq (MACS). *Genome Biol. 9*, 1–9.

(15) Quinlan, A. R., and Hall, I. M. (2010) BEDTools: a flexible suite of utilities for comparing genomic features. *Bioinformatics 26*, 841–842.

(16) Langmead, B., and Salzberg, S. L. (2012) Fast gapped-read alignment with Bowtie 2. *Nat. Methods 9(4)*, 357–359.

(17) Li, B., and Dewey, C. N. (2011) RSEM: Accurate transcript quantification from RNA-Seq data with or without a reference genome. *BMC Bioinformatics 12*, 1–16.

(18) Lawrence, M., Huber, W., Pagès, H., Aboyoun, P., Carlson, M., Gentleman, R., Morgan, M. T., and Carey, V. J. (2013) Software for Computing and Annotating Genomic Ranges. *PLoS Comput. Biol. 9*, e1003118.

(19) Robinson, J. T., Thorvaldsdóttir, H., Winckler, W., Guttman, M., Lander, E. S., Getz, G., and Mesirov, J. P. (2011) Integrative genomics viewer. *Nat. Biotechnol. 29(1)*, 24–26.

(20) Chambers, V. S., Marsico, G., Boutell, J. M., Di Antonio, M., Smith, G. P., and Balasubramanian, S. (2015) High-throughput sequencing of DNA G-quadruplex structures in the human genome. *Nat. Biotechnol. 33(8)*, 877–881.

(21) Huppert, J. L., and Balasubramanian, S. (2005) Prevalence of quadruplexes in the human genome. *Nucleic Acids Res. 33*, 2908–2916.
